# Antioxidation Function of EGCG by Activating Nrf2/HO-1 Pathway in Mice with Coronary Heart Disease

**DOI:** 10.1155/2022/8639139

**Published:** 2022-07-11

**Authors:** Xiaoyi Huang, Yang Chu, Hua Ren, Xiaofen Pang

**Affiliations:** ^1^Department of Geriatrics, Luwan Branch, Ruijin Hospital, Shanghai Jiaotong University School of Medicine, 149 Chongqing South Road Huangpu District, Shanghai 200020, China; ^2^Department of Cardiology, Yueyang Hospital of Integrated Traditional Chinese and Western Medicine, Shanghai University of Traditional Chinese Medicine, Shanghai 200437, China

## Abstract

**Objective:**

To explore the effect and mechanism of epigallocatechin gallate (EGCG) in mice with coronary heart disease (CHD).

**Methods:**

Firstly, a CHD model of mouse was established by feeding mice high-fat diet and randomly divided into four groups, including Model group (0.5% sodium cholate) and 10 mg/kg EGCG, 20 mg/kg EGCG, and 40 mg/kg EGCG groups. After oral administration of sodium cholate or EGCG, HE staining was conducted to assess the pathological changes of mouse cardiac tissues in each group of mice, biochemical kits to measure the levels of blood lipid and oxidative stress substance activity, and western blot to detect matrix metalloproteinase 2 (MMP-2), vascular endothelial growth factor (VEGFA), as well as expression levels of protein related to Nrf2/HO-1/NQO1 pathway in cardiac tissues.

**Results:**

The mice in the CHD model appeared to have myocardial pathological damage with elevated serum total cholesterol (TC), triglyceride (TG), low-density lipoprotein cholesterol (LDL-C), and decreased high-density lipoprotein cholesterol (HDL-C). Of note, administration of EGCG significantly attenuated myocardial injuries and improved blood lipid levels in mice in a concentration-dependent manner. The advent of EGCG significantly decreased the expression of VEGFA and MMP-2 and increased the activity of superoxide dismutase (SOD), when reducing the content of reactive oxygen species (ROS) in the myocardial tissue and upregulating the expression of HO-1, NQO1, and Nrf2.

**Conclusion:**

EGCG may reduce atherosclerotic plaque and alleviate pathological damage in the cardiac tissue of CHD mice as well as improve blood lipid levels with antioxidative effect. The mechanism of its effect may be related to the activation of the Nrf2/HO-1/NQO1 antioxidant pathway in vivo of the CHD mice.

## 1. Introduction

Coronary heart disease (CHD) is a common kind of heart disease in which atherosclerotic pathological changes in the coronary arteries cause vascular obstruction or lumen stenosis, resulting in hypoxia and ischemic necrosis of myocardial tissue and hereby affecting heart function [1]. It is characterized by chest distress and chest pain, which are aggravated after activities and this condition is more prevalent among people over 40 years old, but recent years have witnessed an increased morbidity among young people with the improvement of people's material living standards and lifestyle changes [2]. According to the Chinese Cardiovascular Disease Report 2018, the incidence and mortality of cardiovascular diseases are increasing in China as there are currently 290 million cases, including 11 million with CHD which ranks second in cardiovascular diseases [3]. Therefore, it is essential to find a comprehensive and effective treatment for CHD.

At present, vascular reconstruction, drug therapy, and surgical therapy are the commonly used options for CHD, but they are accompanied with high risk, high cost, complications, drug tolerance, etc. Importantly, traditional Chinese medicines are reported to have significant effects on the treatment of CHD; compatibility or extraction of the active components of Chinese herbal medicines such as *Salvia miltiorrhiza* [4] shows great potential for the prevention and treatment of CHD [5]. Furthermore, Cao Yun systematically reviewed the application of Chinese herbs in CHD [6]. Chinese herbal medicines contain a variety of chemicals, such as alkaloids, glycosides, polyphenols, and terpenoids with various functions including vasodilatation, antioxidation, and antiarrhythmic, analgesic, antibacterial, antiasthmatic, anticancer, anti-Alzheimer's disease activity, etc. [7]. Epigallocatechin gallate (EGCG), the main active ingredient in green tea, is recognized as a promising drug [8]. Previous studies have depicted its various biological effects such as antioxidation, antiapoptosis, anti-inflammation, etc. [9]. In addition, EGCG can also effectively prevent cardiovascular diseases with a protective effect on the heart and blood vessels [10, 11]. As revealed by Chakrawarti et al. in 2016, EGCG, no matter used alone or in combination with other compounds, has ideal effects on the treatment of various types of cancer and metabolic, neurodegenerative, and microbial diseases [12]. However, there is no study about the specific mechanism of action of EGCG in CHD. Based on the above studies, we initially explored the function of EGCG in CHD and the possible mechanism.

Vascular endothelial growth factor (VEGF) family is a regulator of blood vessels and lymphatic vessels, which is mainly studied in the field of cancer and ophthalmology. However, its function can also interfere with the development of atherosclerosis and CHD [13]. In patients with CHD, due to local inflammation, mechanical stress, and cytokines, myocardium will secrete VEGFA, resulting in myocardial deformation, contraction, and recovery damage [14]. Matrix metalloproteinases (MMPs) are a family of zinc dependent proteolytic enzymes. MMPs can regulate various cells and signal pathways responsible for the progression and rupture of atherosclerotic plaques in atherosclerosis. MMP-2 plays an important role in heart and aortic remodeling and is one of the biomarkers to evaluate vascular remodeling [15]. Therefore, we investigated the effect of EGCG on CHD mice by detecting the expression of VEGFA and MMP-2 in the heart tissue of CHD mice.

In this study, a mouse CHD model was established by high-fat diet to detect the effects of EGCG on blood lipids and oxidative stress levels in CHD mice. Besides, our work managed to preliminarily identify the mechanism of action and aimed at providing theoretical basis for the clinical treatment of CHD.

## 2. Materials and Methods

### 2.1. Laboratory Animal

Thirty 16-week-old female ApoE −/− mice (18 g–22 g) were purchased from Shanghai Southern Model Biology Center and subjected one-week adaptive feeding (24 ± 1°C, 65% relative humidity, 12 h light/dark cycle, food and water available freely). The study was approved by the Guangdong Medical Laboratory Animal Center Animal Research Ethics Committee (C202205-9) and conducted in accordance with the approved guidelines.

### 2.2. Grouping and Treatment

A total of 30 16-week-old female ApoE −/− mice (18 g–22 g) were randomly divided into 5 groups (*n* = 6, each group): Control group, Model group, and 10 mg/kg EGCG, 20 mg/kg EGCG, and 40 mg/kg EGCG groups. The animals in the Control group were fed with conventional diet, and the other mice were fed high-fat diet (containing 1.25% cholesterol and 0.5% sodium cholate) to induce CHD. With the mice in Model group untreated, those in EGCG group were orally administered 10, 20, and 40 mg/kg/day of EGCG for six weeks, respectively. One hour after the last administration, serum, aorta, and cardiac tissues were collected from the mice for subsequent experimental tests.

### 2.3. Hematoxylin and Eosin (HE) Staining

Myocardial tissue sections from mice were dried and fixed at room temperature for 30 s, washed with 1  ×  PBS for 2 s, and stained with hematoxylin (60°C) for 60 s. After washing with 1  ×  PBS for 10 s, the sample was differentiated with 1% hydrochloric acid alcohol for 3 s, washed, stained with eosin for 3 min, and dehydrated with 70%, 80%, and 95% ethanol and absolute ethanol for 5 min and xylene three times/5 min. Finally, the sections were cleared with gum, and the pathological changes in myocardial tissue were observed under a light microscope [9].

### 2.4. Detection of Biochemical Indicators in Mice

Blood samples were taken from mice and centrifuged to collect serum. By using biochemical kits (Nanjing Jiancheng Bioengineering Institute Co., Ltd.), the levels of total cholesterol (TC, A111-1-1), triglyceride (TG, A110-1-1), high-density lipoprotein cholesterol (HDL-C, A112-1-1), and low-density lipoprotein cholesterol (LDL-C, A113-1-1) in mice serum were measured in strict accordance with the kit instructions.

Mouse cardiac tissue was taken and mixed with PBS (pH 7.4). Then, the sample was fully homogenized by a homogenizer and centrifuged at 2000–3000 r/min for 20 min, and the supernatant was collected, and the contents of SOD activity (A001-3-2) and ROS (E004-1-1) in mouse cardiac tissue were measured by a biochemical kit (Nanjing Jiancheng Bioengineering Institute Co., Ltd.).

### 2.5. Western Blot Assay

Proteins were extracted from cardiac tissue with radio immunoprecipitation assay (RIPA) lysis buffer (P0013 B, Beyotime) and their concentrations were determined with a bicinchoninic acid (BCA) protein kit (P0010S, Beyotime). The protein samples were separated with sodium dodecyl sulfate-polyacrylamide gel electrophoresis (SDS-PAGE) gels and transferred to polyvinylidene fluoride (PVDF) membranes. The membrane was blocked with 5% skim milk powder in TBS + tween (TBST) for 1 h and probed with primary antibodies (GAPDH, ab181602, Abcam; MMP-2, ab37150, Abcam; VEGFA, ab46154, Abcam; Nrf2, ab92946, Abcam; HO-1, ab13243, Abcam; NQO1, ab80588, Abcam) overnight at 4°C and shaken and rinsed three times with TBST solution. Then, the blot was incubated with diluted secondary antibodies (horseradish enzyme-labeled goat antirabbit IgG (*H* + *L*), ZB-2301, Chinese fir golden bridge) for 2 h. After washing, enhanced chemiluminescence (ECL) luminescence agent (P0018AS, Biyuntian) was evenly instilled. Finally, the FliorchemHD2 imaging system was used for scanning, analyzing, and collecting images as well as measuring the gray values of each band through Image *J*.

### 2.6. Statistical Analysis

Data were analyzed by SPSS 21.0 software and presented as expressed as mean ± standard deviation (Mean ± SD). *T*-test was used for the comparison between two groups and one-way analysis of variance for comparison of multiple groups. *P* < 0.05 indicates statistical significance.

## 3. Results

### 3.1. EGCG Alleviates Cardiac Pathological Damage in CHD Mice

**Figure 1:**
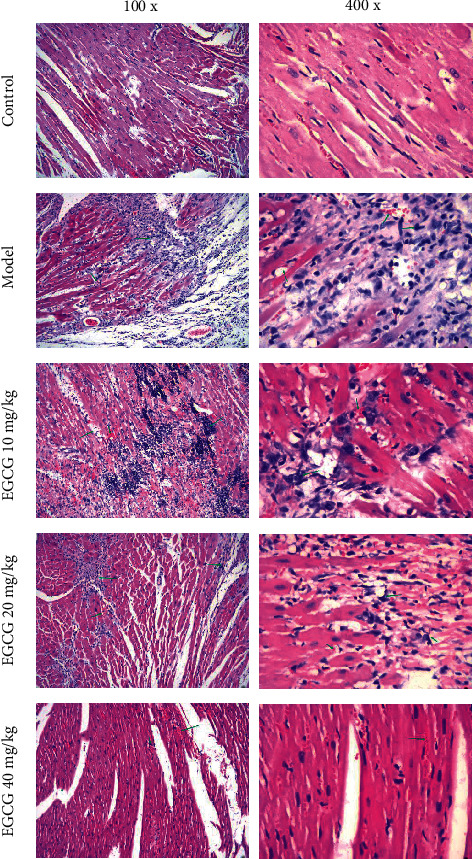
Effect of EGCG on cardiac pathological damage in CHD mice. The green arrow indicates the lesion site of the cardiac tissue in CHD mice.

In this study, the cardiac pathological damage in mice was first assessed by HE staining. The results showed clear depiction of aortic sinuses in the hearts of mice in the control group without any plaques, while atherosclerotic lesions were observed in the Model and EGCG groups with the disintegration, fragmentation, or loss of myocardial fibers. Of note, compared with the Model group, after EGCG treatment, the myocardial structure was significantly improved, and the myocardial fibers were orderly arranged without significant fragmentation. It indicated that the cardiac pathological injuries of mice treated with EGCG were relieved to varying degrees with the increase of EGCG concentration (Figure 1). This phenomenon shows that EGCG might ameliorate myocardial injuries in mice.

### 3.2. EGCG Improves Lipid Levels in CHD Mice

**Figure 2:**
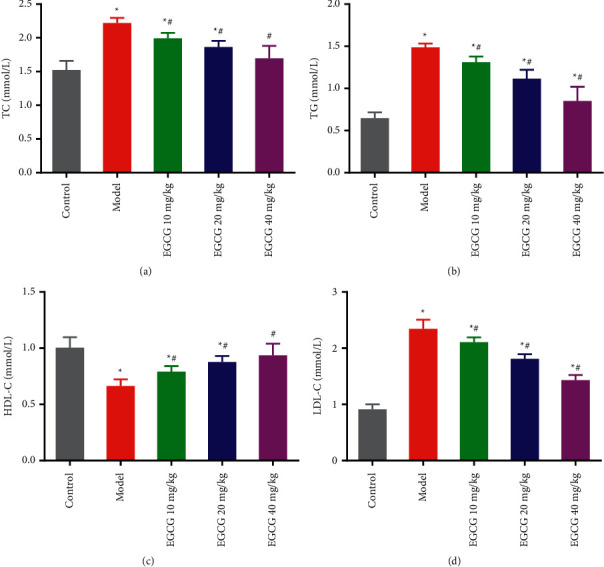
Effect of EGCG on lipid levels in vivo in CHD mice. ELISA of the serum levels of TC (a), TG (b), HDL-C (c), and LDL-C (d) in each group. ^*∗*^*P* *<* 0.05 *vs.* Control group; ^#^*p* < 0.05 *vs.* Model group. CHD, coronary heart disease; EGCG, epigallocatechin gallate; TC, total cholesterol; TG, triglyceride; HDL-C, high-density lipoprotein cholesterol; LDL-C, low-density lipoprotein cholesterol.

Previous studies have showed that dyslipidemia promotes the progression of CHD [16]. In our study, the blood lipid levels of mice in each group were measured with biochemical kits (Figures 2(a)–2(d)). It is found that compared with the Control group, the serum levels of TC, TG, and LDL-C were significantly increased in the Model and EGCG groups, and the level of HDL-C was decreased (*P* < 0.05). However, administration of EGCG dose-dependently elevated the levels of HDL-C and decreased the serum levels of TC, TG, and LDL-C in CHD mice (*P* < 0.05). These results suggest that EGCG can significantly improve the lipid environment to hinder the progression of CHD in mice.

### 3.3. EGCG Alleviates Oxidative Damage of Cardiac Tissues in CHD Mice

**Figure 3:**
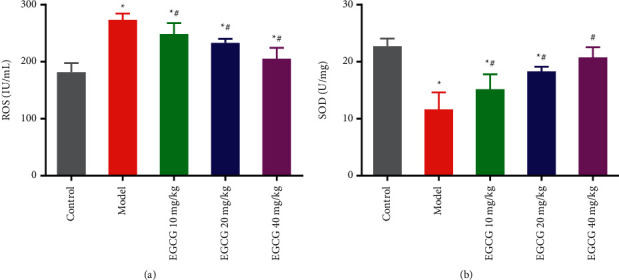
Effect of EGCG on oxidative stress in vivo in CHD mice. (a) The activity level of ROS in cardiac tissues of mice in each group was detected biochemically; (b) the activity level of SOD in cardiac tissues of mice in each group was detected biochemically. ^*∗*^*p* < 0.05 vs. Control group; ^#^*p* < 0.05 vs. Model group. CHD, coronary heart disease; EGCG, epigallocatechin gallate; SOD, superoxide dismutase, ROS, reactive oxygen species.

In CHD, excessive ROS production will not only lead to oxidative imbalance in the body but also promote the occurrence and development of CHD, and the antioxidant enzyme SOD can reduce the risk of CHD [17]. By measuring the activity of ROS and antioxidant SOD in cardiac tissues of mice in each group, the antioxidant effect of EGCG can be assessed. The results showed that ROS activity was significantly increased while SOD activity was significantly decreased in cardiac tissues of Model mice, compared with the Control group. After treating with EGCG, ROS activity was significantly decreased and SOD activity was significantly increased in a concentration-dependent manner (Figures 3(a)–3(b)). Based on the above results, it indicated that EGCG is able to alleviate the oxidative damage of cardiac tissues in mice with CHD by decreasing ROS activity and increasing SOD activity.

### 3.4. Effect of EGCG on the Progression of Atherosclerosis in CHD Mice

**Figure 4:**
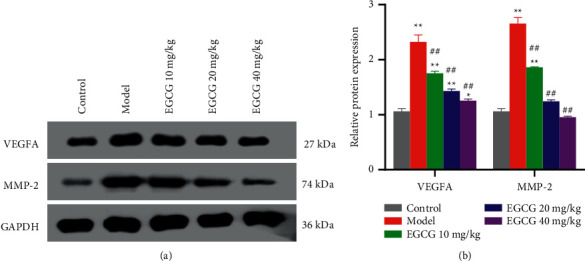
Effect of EGCG on VEGFA and MMP-2 expression levels in cardiac tissues of mice. (a, b) Western blot analysis of the protein levels of VEGFA and MMP-2 in cardiac tissues of mice in each group and corresponding quantification of the protein expression. ^*∗∗*^*p* *<* 0.01 *vs.* Control group; *^##^p* < 0.01 *vs.* Model group. CHD, coronary heart disease; EGCG, epigallocatechin gallate; VEGFA, vascular endothelial growth factor; MMP-2, matrix metalloproteinase 2.

Using western blot analysis, we further detected the protein expression levels of MMP-2 and VEGFA, and found elevation of VEGFA and MMP-2 levels in the cardiac tissues of CHD mice (Figures 4(a) and 4(b)). However, compared with the Model group, EGCG groups showed decreased expression levels of VEGFA and MMP-2 in cardiac tissues (*P* < 0.01) with the increased concentration of ECGC following more prominent decline (Figure 4). The above results indicated that EGCG might inhibit atherosclerosis in mice with CHD.

### 3.5. EGCG Activates the Nrf2/HO-1/NQO1 Pathway in CHD Mice

**Figure 5:**
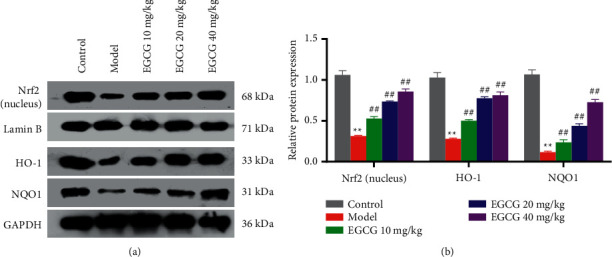
Effect of EGCG on Nrf2/HO-1/NQO1 pathway in mice with CHD. (a, b) The protein levels of Nrf2, HO-1, and NQO1 in cardiac tissues of mice in each group were detected by western blot; ^∗∗^*p* < 0.01 vs. Control group; ^##^*p* < 0.01 vs. Model group. CHD, coronary heart disease; EGCG, eepigallocatechin gallate.

Nrf2 is the core factor of antioxidant system. EGCG is a powerful Nrf2 agonist, which can activate Nrf2 antioxidant signaling pathway and initiate the expression of downstream antioxidant genes heme oxygenase 1 (HO-1) and reduced coenzyme/quinone oxidoreductase 1 (NQO1) [18, 19]. In order to explore whether EGCG affects the activation of Nrf2/HO-1 in CHD mice, a major oxidative stress pathway in vivo, the expression levels of related protein Nrf2, HO-1, and NQO1 in this pathway were detected. With decreased expressions of NQO1 and Nrf2, and HO-1 observed in the Model group, the addition of EGCG, especially at the concentration of 40 mg/kg, restored their expressions in the cardiac tissues (*P* < 0.01) (Figures 5(a) and 5(b)). The above results indicated that EGCG can activate the Nrf2/HO-1/NQO1 pathway in CHD mice by upregulating the protein expression of HO-1, NQO1, as well as nuclear Nrf2.

## 4. Discussion

Studies showed that the increase of LDL-L, the decrease of HDL-C in serum, and the functional impairment of vascular endothelial cells are the initial links to stimulate the development of atherosclerosis, and in the process of endothelial cell migration, VEGF plays an important role [11, 20]. Oxidative stress can lead to an increase in oxygen free radicals in arterial endothelial cells and a decrease in the activity of enzymes that scavenge oxygen free radicals, such as SOD. As a result, the oxygen free radicals accumulate and react with unsaturated fatty acids on the cell membrane to produce MDA, which further destroys the structure and function of the cell membrane [21]. At the same time, extracellular matrix metalloproteinase inducer (EMMPRIN) can induce the release of MMP-2 and thus induce the processes of atherosclerotic plaque formation, sclerosis, progression, rupture, etc. [22]. Therefore, in this study, lipid levels, SOD activity, ROS activity, VEGFA, and MMP-2 expression were measured in mice with CHD to assess the effect of EGCG on the formation as well as progression of atherosclerosis.

EGCG is the main component of green tea catechins. By inhibiting the aggregation of platelets and the production of TXB2 and P-selectin, EGCG may inhibit the activation and aggregation of blood platelets when exerting the antithrombotic function [23, 24]. In recent years, the significance of EGCG to lipid metabolism has been elucidated. EGCG reduces blood lipids by regulating the synthesis of triglycerides, cholesterol, fatty acids (FA), high-density lipoprotein, low-density lipoprotein, etc., or promoting the catabolism of body fat [25, 26]. Basiricò et al. found that EGCG could play an antioxidative stress role by efficiently scavenging free radicals and chelating with metal ions [27], or regulating the expression of SOD and ROS levels in vivo [28, 29]. The experiments in this study also found that EGCG not only significantly reduced the red lipid plaques in the aorta but also attenuated myocardial injuries in CHD mice. Besides, administration of EGCG effectively decreased blood lipids and improved the blood lipid environment of CHD mice by downregulating TC, TG, and LDL-C and upregulating HDL-C in serum. At the same time, EGCG was also found to play an antioxidant role by inhibiting ROS production and promoting SOD expression.

The interaction between Nrf2, a key transcription factor against oxidative stress, and the antioxidative response element can initiate the transcription and expression of downstream antioxidant enzyme genes and then scavenges ROS produced by the body enhancing the antioxidant capacity of the body [30]. NQO1, as one of the Nrf2 downstream target genes, is considered as the major antioxidant enzyme [31]. Increased NQO1 expression is indicated to prevent hyperoxia-induced lung injury [32]. Feng et al. showed that Nrf2/HO-1/NQO1 signaling pathway is one of the important mechanisms for the body's beneficial effects against oxidative/stress injury [33].

Oxidative stress reaction is the main influencing factor of coronary atherosclerosis, which is closely related to the occurrence and development of angina pectoris [21]. Myocardial ischemia in patients with CHD will produce a large number of oxygen free radicals, resulting in the imbalance of oxygen free radical content in the body [34]. EGCG can scavenge oxygen free radicals, relax vascular endothelial cells, increase antioxidant capacity, and significantly improve oxidative stress response. Notably, our work elucidates that EGCG can activate the Nrf2/HO-1/NQO1 pathway by upregulating the expression of HO-1, NQO1, and Nrf2, thereby reducing oxidative stress injury, which is consistent with other study [19]. However, only the activation of Nrf2/HO-1/NQO1, a major pathway of oxidative stress, was examined in our study. Whether EGCG acts through other pathways remains unclear. In addition, our study subjects are mice and have not been clinically validated. Therefore, a large number of clinical studies are needed for validating function of EGCG in human with CHD.

## 5. Conclusion

To sum up, EGCG can not only alleviate the pathological damage of myocardial tissue in mice with CHD but also reduce the levels of blood lipid and ROS as well as enhance the body's antioxidant capacity in a concentration-dependent manner. The above effects may be related to the activation of the antioxidant pathway Nrf2/HO-1/NQO1 in mice with CHD.

## Data Availability

The data used to support the findings of this study are available from the corresponding author upon request.
